# Rational design on high-performance triboelectric nanogenerator consisting of silicon carbide@silicon dioxide nanowhiskers/polydimethylsiloxane (SiC@SiO_2_/PDMS) nanocomposite films

**DOI:** 10.1186/s11671-023-03822-8

**Published:** 2023-04-21

**Authors:** Kun Zhao, Wanru Sun, Suixin Li, Zhenhua Song, Ming Zhong, Ding Zhang, Bing-Ni Gu, Ming-Jin Liu, Hao Fu, Hongjie Liu, Cheng Meng, Yu-Lun Chueh

**Affiliations:** 1 State Key Laboratory of Advanced Processing and Recycling of Nonferrous Metals, Lanzhou University of Technology, Lanzhou, 730050 People’s Republic of China; 2 School of Materials Science and Engineering, National Institute for Advanced Materials, Nankai University, Tianjin, 300350 People’s Republic of China; 3 Department of Materials Science and Engineering, National Tsing Hua University, Hsinchu, 30013 Taiwan; 4 School of Chemistry and Chemical Engineering, Guangxi University, Nanning, 530004 People’s Republic of China; 5 Colleage of Semiconductor Research, National Tsing-Hua University, Hsinchu, 30013 Taiwan; 6 Department of Physics, National Sun Yat-Sen University, Kaohsiung, 80424 Taiwan; 7 Frontier Research Center on Fundamental and Applied Sciences of Matters, National Tsing Hua University, Hsinchu, 30013 Taiwan; 8 Jiangxi Province Key Laboratory of Polymer Micro/Nano Manufacturing and Devices, School of Chemistry Biology and Materials Science, East China University of Technology, Nanchang, 330013 People’s Republic of China

**Keywords:** SiC@SiO_2_ nanowhiskers, SiC@SiO_2_/PDMS composite films, Chemical modified, Superhydrophobic, Triboelectric nanogenerator

## Abstract

The relatively low output performance of triboelectric nanogenerator (TENG), which faces a challenge in performance improvement, limits its practical applications. Here, a high-performance TENG consisting of a silicon carbide@silicon dioxide nanowhiskers/polydimethylsiloxane (SiC@SiO_2_/PDMS) nanocomposite film and a superhydrophobic aluminum (Al) plate as triboelectric layers is demonstrated. The 7 wt% SiC@SiO_2_/PDMS TENG presents a peak voltage of 200 V and a peak current of 30 μA, which are ~ 300 and ~ 500% over that of the PDMS TENG, owing to an increase in dielectric constant and a decrease in dielectric loss of the PDMS film because of electric insulated SiC@SiO_2_ nanowhiskers. Furthermore, a 10 μF capacitor can be charged up to 3 V within ~ 87 s, which can be continuously operated on the electronic watch for 14 s. The work provides an effective strategy for improving output performance of TENG by adding core–shell nanowhiskers to modulate the dielectric properties of organic materials.

**Graphical abstract:**

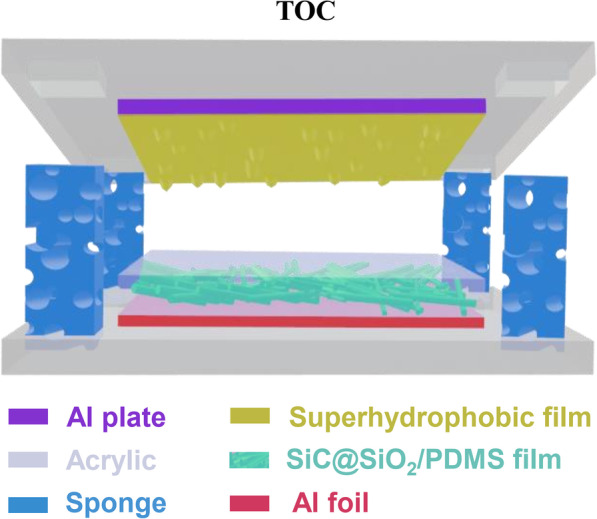

**Supplementary Information:**

The online version contains supplementary material available at 10.1186/s11671-023-03822-8.

## Introduction

Triboelectrification is a common phenomenon in daily life, which is often thought to have negative effects that are managed to be ignored. In 2012, Wang et al*.* first invented a triboelectric nanogenerator (TENG) as a novel energy harvester that can convert various mechanical energies in the surrounding environment into electrical energy based on the coupling of triboelectrification and electrostatic induction [1–3]. TENGs have already captured increasing attention, not only possessing the advantages of simple preparation, wide material selection, lightweight, low cost, and efficient harvesting of low-frequency energy but also providing a new energy supply method for nano/micro-electronic devices [4–9]. Many reports proved that TENG has been widely used in self-powered sensing [10, 11], portably wearable electronics [12], micro-nano energy [13–15], blue energy [16, 17] and high voltage power [18]. However, the relatively low output current and power of the TENG significantly limit its practical application. Therefore, the enhanced output performance of TENGs becomes a necessary and challenging task.

The inherent characteristics of triboelectric materials are closely related to the output performance of TENGs, which is one of the effective ways to enhance triboelectric charge density by optimizing the dielectric properties of triboelectric materials. The output performance can be efficiently improved by increasing the contact area of the material surface [19, 20], charge injection [21, 22], surface self-assembly treatment [23, 24], and incorporation of high dielectric particles [25–28]. Despite these improvements, it still remains a challenge to develop triboelectric materials with higher surface charge density. Inspired by the first report of a piezoelectric nanogenerator with semiconductor zinc-oxide (ZnO) nanowire arrays by Wang et al. in 2006 [29]. Sun et al*.* [30] realized great improvement in output voltage and current from nanogenerator by adding ZnO nanoparticles and multiwall-carbon nanotubes into polydimethylsiloxane (PDMS). The difference in electrical conductivity between semiconductor fillers and polymer matrix at the heterogeneous interface leads to interfacial polarization, resulting in the high dielectric constant in the composite film. However, semiconductor fillers inevitably bring in masses of free charges, resulting in an increase in electrical leakage current and dielectric loss [31–34]. It can efficiently reduce dielectric loss by introducing an insulating layer coating on the semiconductor fillers to form a core–shell structure. It is predicted that the introduction of silicon dioxide (SiO_2_) shell with excellent insulation performance by thermal oxidation treatment on silicon carbide (SiC) whiskers can not only improve the dispersity of the filler in the polymer matrix but also effectively reduce the dielectric loss by inhibiting the carrier migration in the composites [35–39].

In this regard, we report a hydrophobic and high-performance TENG with the vertical contact-separation mode. A superhydrophobic aluminum (Al) plate and a SiC@SiO_2_/PDMS nanocomposite film are used as positive and negative triboelectric layers. The dielectric constant and dielectric loss of the PDMS composite films can be modulated by adjusting the doping concentrations of SiC@SiO_2_ nanowhiskers and the triboelectric charge on the material surface can be effectively improved. Furthermore, the strong hydrophobicity of positive and negative triboelectric layers leads to the self-cleaning performance of the TENG. The output performance of the fabricated TENG with various mass fractions of SiC@SiO_2_ nanowhiskers under different working conditions have systematically studied and optimized. By adding 7 wt% SiC@SiO_2_ nanowhiskers into the PDMS matrix, the dielectric constant and dielectric loss of the composite film are 2.83 and 0.0023, which are 25.2% higher and 37.8% lower than that of pure PDMS film. The output voltage and current of the TENG are as high as 200 V and 30 μA, corresponding to an increase by 300% and 500% compared with pure PMDS film-based TENG, respectively. The optimized TENG delivers a maximum output power of approximately 0.68 mW at a load of 1.3 MΩ, which can be the power supply of the electronic watch with a continuously charged period of ~ 14 s, exhibiting a great promise in practical applications.

## Material and methods

### Preparation of SiC@SiO_2_ core–shell nanowhiskers and SiC@SiO_2_/PDMS nanocomposite films

First, the commercial raw SiC whiskers were washed with water and ethanol to remove impurities that can be adsorbed on the surface of the nanowhiskers. Second, the washed SiC whiskers were calcined at 700 °C for 3 h in a tube furnace under an air atmosphere to obtain core–shell SiC@SiO_2_ whiskers [40]. To prepare various SiC@SiO_2_/PDMS nanocomposite films, the SiC@SiO_2_ nanowhiskers with different weight percentages from 0 to 11 wt% were dispersed into a PDMS matrix. Note that the PDMS precursor and curing agent with a weight ratio of 10:1 were used. Then, the uniform SiC@SiO_2_/PDMS mixture was coated on a flat Al foil through a blade coater with a thickness of 200 μm. Third, the Al foil was horizontally installed in an oven at 85 °C for 2 h, and the homogeneous SiC@SiO_2_/PDMS nanocomposite films with an average thickness of 120 μm can be obtained.

### Preparation of superhydrophobic surface on an Al plate

Al plates were polished and ultrasonically cleaned in alcohol and washed with deionized water and were then chemically etched for 60 s in a mixed solution (hydrochloric acid (HCl) and deionized water (H_2_O) with a volume ratio in 2:3) at room temperature. Then, the etched Al plates were ultrasonically washed with deionized water and ethanol, then placed in an oven at 110 °C for 30 min. The cleaned and etched Al plates were further modified in octadecyltrichlorosilane (OTS)/toluene solution at room temperature for 48 h. After removal from the solution, the samples were washed with alcohol and heated at 130 °C for 2 h.

### Fabrication of TENG

Acrylic plates were cut into two 60 × 60 mm quadrates as the support plates. A modified Al plate with the dimensions of 40 × 40 × 0.46 mm was attached to an acrylic plate as an electrode, and the prepared superhydrophobic surface of the Al plate acts as the positive triboelectric layer. For a negative triboelectric layer, SiC@SiO_2_/PDMS nanocomposite film with the dimension of 40 × 40 × 0.2 mm was covered with an Al foil-covered acrylic plate. Subsequently, four sponges were employed as the buffer layer between two acrylic substrates. Two copper wires were connected to two Al electrodes for the electrical connection.

### Characterization and measurements

The morphologies of the sample were measured by a scanning electron microscope (SEM, SU5000) and a transmission electron microscope (TEM, JEM-F200). The crystal structure of SiC and SiC@SiO_2_ whiskers were characterized by X-ray diffraction (XRD, D8 ADVANCE), using Cu Kα radiation. The surface elements from different samples were detected by X-ray photoelectron spectroscopy (XPS, AXIS SUPRA). Contact angles were measured by an optical contact angle meter (DSA 100) at room temperature. The dielectric properties were characterized by a broadband dielectric impedance spectrometer (Novocontorl concept 90) at room temperature. The forces were supplied by a liner motor (LinMot 1100). The force values were measured using a micro pressure sensor (AT8106). The voltage and current signals of the TENG were recorded by a digital oscilloscope (Tektronix MDO3014) and a system electrometer (Keithley 2611B), respectively. The transfer charge of the TENG was characterized using a Keithley electrometer (Keithley 6514).

## Results and discussions

**Fig. 1:**
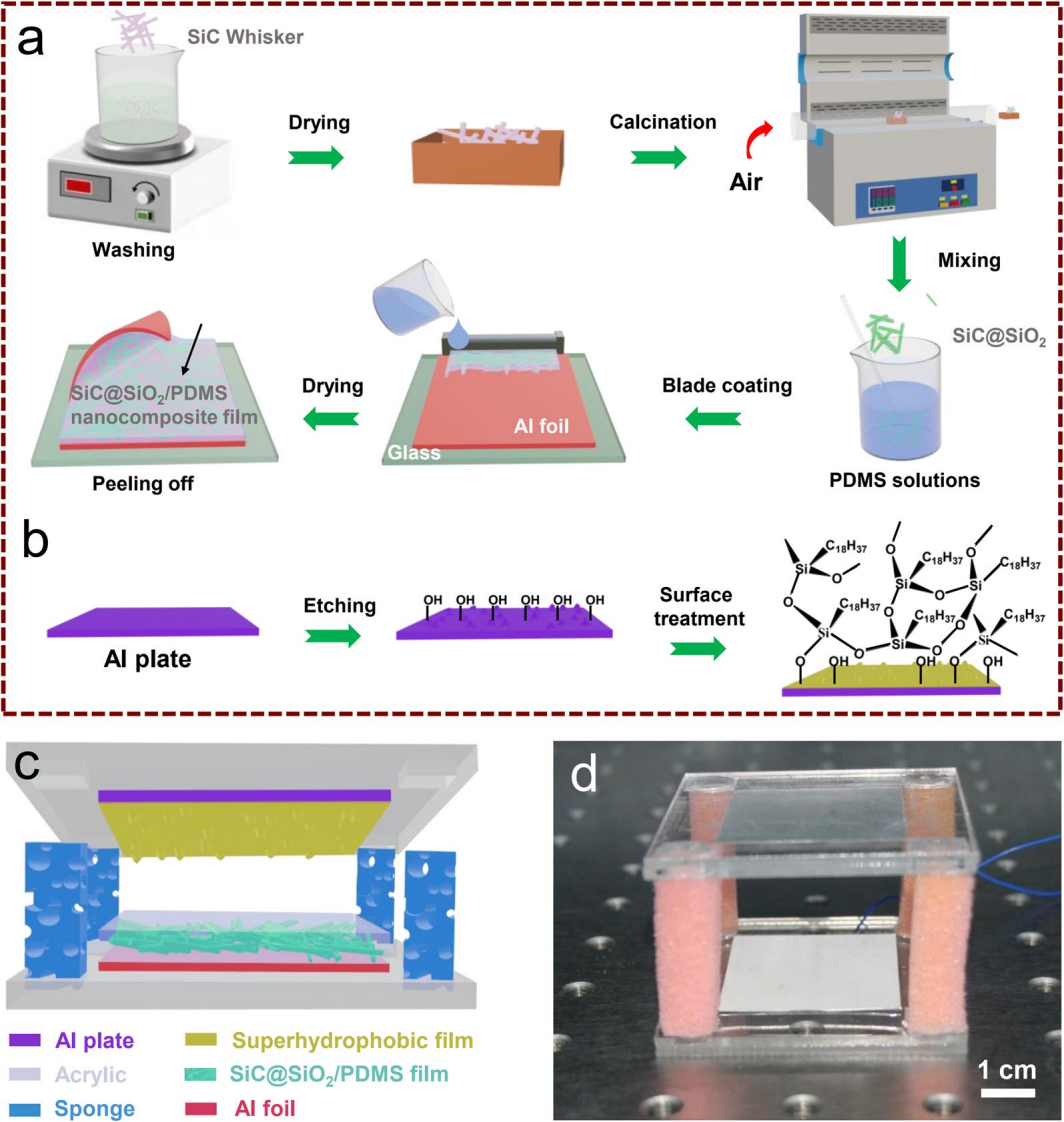
**a**, **b** Schematic showing the preparation of SiC@SiO_2_/PDMS composite films (**a**) and etched Al plate modified with octadecyltrichlorosilane (**b**). **c** Structure diagram for the TENG based on the SiC@SiO_2_/PDMS composite films and superhydrophobic layer. **d** Photograph of the as-fabricated TENG

Figure 1a shows the fabrication process of the composite film and the detailed steps were described in the experimental section. After the air calcination, the SiC@SiO_2_ core–shell whiskers can be finally obtained because the SiC surface is thermally oxidized to SiO_2_. Subsequently, the SiC@SiO_2_ whiskers were added to the PDMS matrix and were stirred for 30 min to gain a homogeneous SiC@SiO_2_/PDMS mixed solution. After the blade coating and the thermal treatment, SiC@SiO_2_/PDMS nanocomposite film with a thickness of 200 μm was successfully prepared as a negative triboelectric material. As displayed in Fig. 1b, one surface of a 0.5 mm-thick Al plate was firstly etched with HCl-H_2_O solution to form many hydrophilic hydroxyl groups, and then was modified by a mixture solution of OTS and toluene, exhibiting superhydrophobic performance with many hydrophobic molecular groups on the Al surface. The main reason is that the surface energy of Al sheet is effectively reduced after being modified by silane, which makes it shows superhydrophobicity. Figure 1c and d shows the corresponding structural diagram and optical image of a TENG, respectively. A superhydrophobic Al plate and SiC@SiO_2_/PDMS nanocomposite film with the Al electrode are fixed on an acrylic plate as positive and negative triboelectric layers as shown in Fig. S1. Moreover, the four rectangular column sponges act as the supporting framework and can realize the role of contact and separation.

**Fig. 2:**
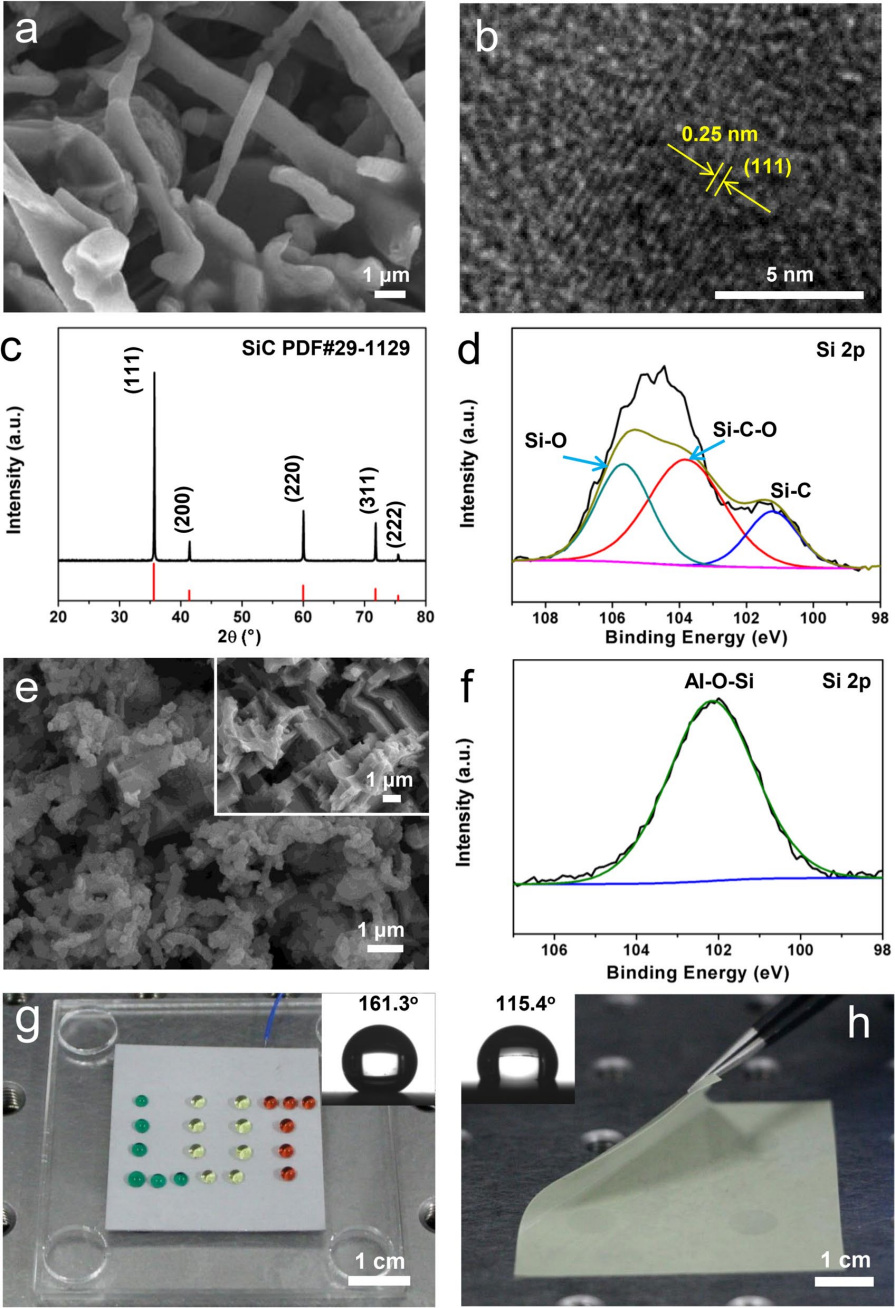
**a**, **b** High-magnification SEM (**a**) and TEM (**b**) images of SiC@SiO_2_ core–shell nanowhiskers. **c** XRD patterns of SiC nanowhiskers. **d** High-resolution Si 2*p* XPS spectrum of SiC@SiO_2_ nanowhiskers. **e** SEM images of the etched (the insert) and modified Al electrodes. **f** High-resolution Si 2*p* XPS spectrum of the modified Al electrode. **g** Photograph of water droplets with "LUT" pattern on the as-prepared superhydrophobic surface of Al plate (insert: the sample with a contact angle of about 161.3°). **h** Photograph of flexible SiC@SiO_2_/PDMS composite film (insert: the sample with a contact angle of about 115.4°)

Figures 2a and S2a show the scanning electron microscope (SEM) images with different magnifications of core–shell SiC@SiO_2_ nanowhiskers after the calcination of SiC whiskers. Clearly, the short rod-shaped SiC@SiO_2_ nanowhiskers of approximately 500–950 nm in diameter and over 10 μm in length can be obtained. According to the further characterization of the transmission electron microscope (TEM), the core–shell structure of SiC@SiO_2_ nanowhiskers can be observed as shown in Fig. S2b, with which the thickness of the SiO_2_ shell is approximately 15–20 nm. Note that the SiO_2_ in the outer layer of the core–shell is an amorphous phase, and the inter-planar spacing between two adjacent lattice fringes in the inner layer of the core was found to be 0.25 nm (Fig. 2b), corresponding to the (111) plane of the SiC crystal (Fig. 2c). Other main diffraction peaks of SiC@SiO_2_ shell at 2*θ* = 35.7°,42.5°,60.2°,72.0°, and 73.8° are in good agreement with the standard SiC peak (JCPDS card no.29–1129), confirming the cubic crystal structure of the SiC core. XPS was used to verify the existence of the SiC@SiO_2_ and the element content change after and before the calcination process. Figures 2d, S3, and S4 show XPS spectra of SiC nanowhiskers after and before the calcination process. Obviously, C 1*s*, O 1*s*, Si 2p and Si 2*s* peaks were found in full spectra (Figs. S3a and S4a). According to the high-resolution XPS spectra of Si 2*p* in SiC@SiO_2_ and SiC (Figs. 2d and S3b), the percentage of Si–C peak area at 101.2 eV in SiC@SiO_2_ greatly decreases, while all percentages of Si–C–O and Si–O at 102.5 and 103.5 eV increase. Clearly, the peak areas of C-O and C = O at 285.8 and 286.7 eV in SiC@SiO_2_ are significantly increased, while the percentage of Si–C peak area is reduced compared with that of the SiC. Note that the C 1*s* at 284.6 eV was the reference for binding energy calibration. (Figs. S3c and S4b). The peak at 532.4 eV referring to the O 1*s* is from the air (Fig. S3d), while the peak at 534.0 eV corresponds to the Si–O bond (Fig. S4c). The comprehensive XPS spectra of the Si 2*p*, C 1*s* and O 1*s* confirm the occurrence of thermal oxidation owing to the formation of SiO_2_ layer on the surface of SiC whiskers. Furthermore, the surface of the Al plate etched by HCl–H_2_O solution presents a “step-like” morphology with a scale of 200 ~ 500 nm (Fig. 2e), which increases the surface roughness and provides the necessary for the next step to build a superhydrophobic surface. Moreover, there are still many “step-like” and “curls” with smaller microstructures on the obtained superhydrophobic surface modified by the octadecyltrichlorosilane/toluene solution, namely the silane-modified Al plate. The corresponding XPS spectra of the etched Al plate and the silane-modified Al plate are shown in Figs. S5 and S6, respectively. The full spectra of the etched Al plate confirm the appearance of C 1*s*, O 1*s* and Al 2*p* peaks (Fig. S5a). However, the Si 2*p* and Cl 2*p* peaks are displayed in the XPS full spectrum of the silane-modified Al plate (Fig. S6a). The presence of Al–O–Si with a binding energy of 102.2 eV proves the occurrence of functionalized reaction on the surface of the etched Al plate treated by hydroxyl and silane modifier (Fig. 2f). Figures S5b and S6b are the high-resolution XPS spectra of C 1*s* (C–C, C–O, and C = O located at 284.6, 286.2 and 288.3 eV, respectively) of the etched Al plate and the silane-modified Al plate, and the C–C peak at 284.6 eV as a reference to compensate the charging effects. Figures S5c and S6c are the high-resolution XPS spectra of O 1*s* (Al–OH and Al–O–Si are located at 531.2 and 532.1 eV, respectively). The high-resolution XPS spectrum of Al 2p at 73.8 eV for the etched Al plate as shown in Fig. S5d corresponds to Al–OH. Two peaks located at 76.0 and 74.2 eV are attributed to the AlCl_3_ and Al–OH for the modified Al plate (Fig. S6d), for which the binding energies at 197.5 and 199.2 eV are Cl 2p_3/2_ and Cl 2p_1/2_ of AlCl_3_, respectively (Fig. S6e). Clearly, the silane can be successfully modified on the surface of the etched Al plate, which cannot only effectively reduce the surface energy of the Al plate to make it having excellent superhydrophobic property, but also enhance the charge trapping ability, which is critical to improve the moisture resistance and output performance of the device. To evaluate the hydrophobicity after the silane-modified Al plate, the contact angles of the sample were measured as shown in Fig. 2g. A water droplet on the silane-modified Al plate keeps a perfectly spherical shape as shown in the inset of Fig. 2g with an average contact angle of ~ 161.3°, which is larger than that of the etched Al plate (83.7°) (Fig. S7a). Furthermore, the excellent superhydrophobic performance can be demonstrated by creating a pattern of the initials of LUT (Lanzhou University of Technology) from water droplets on the silane-modified Al plate. The excellent superhydrophobic performance is mainly caused by a stable Cassie model owing to the combined effects of the micro-nano structure formed on the etched surface of Al plate associated with the low surface energy by the silane modification [41]. In addition, the average contact angle on the surface of SiC@SiO_2_/PDMS nanocomposite film is about 115.4° (Fig. 2h), revealing good hydrophobicity, intrinsically and no obvious variation compared with the contact angle of the pure PDMS film, which is about 110.4° (Fig. S7b). The above analyses prove that the SiC@SiO_2_ core–shell nanostructure still retains the original morphology of the original SiC whiskers and SiC@SiO_2_ whiskers may not affect the hydrophobicity of the PDMS film.

**Fig. 3:**
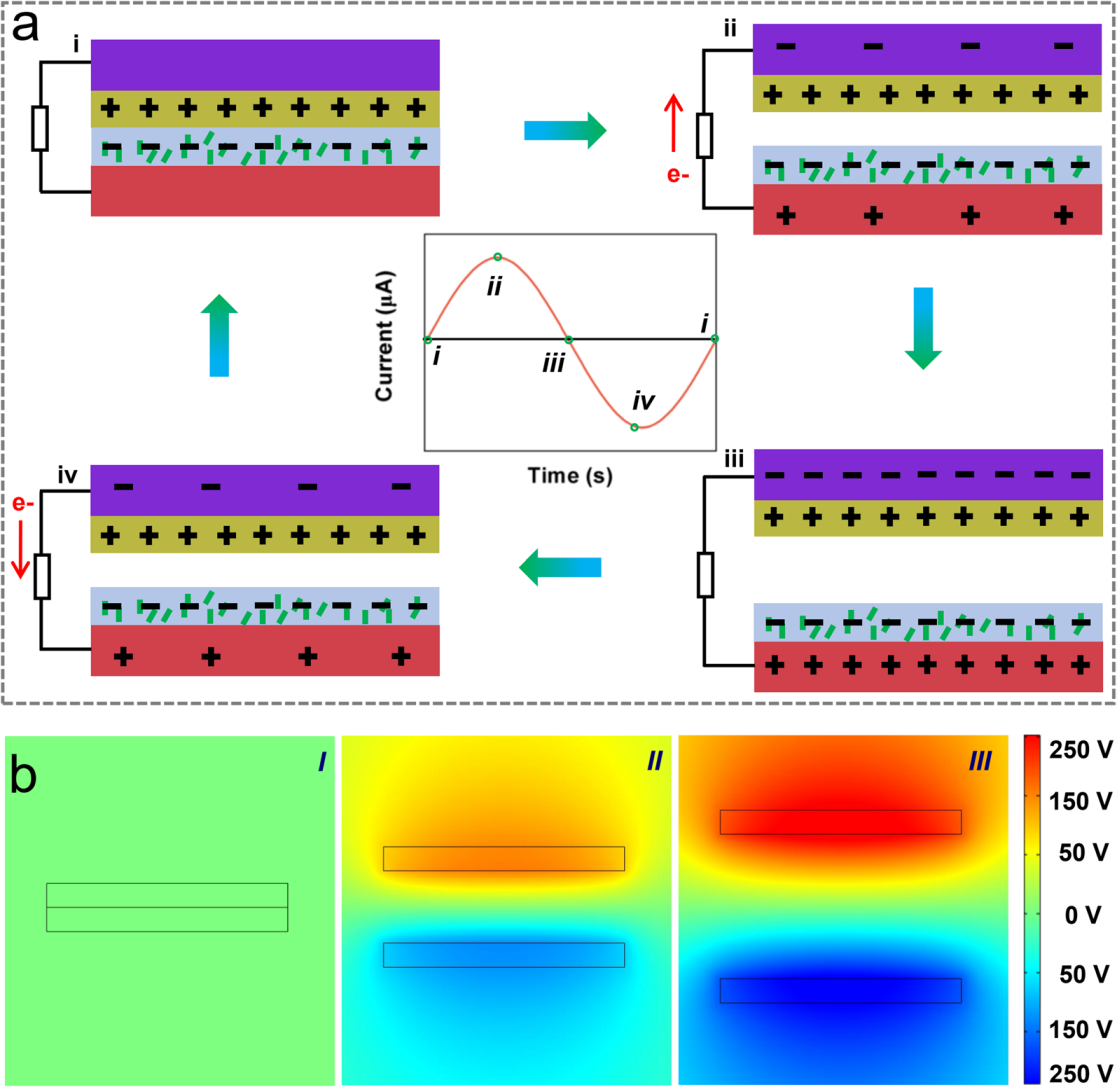
**a** Working mechanism of the TENG. **b** Simulations of electric potential distributions during the contact-separation mode of the TENG at each step (I-III) by COMSOL Multiphysics

To realize high triboelectric output performance, nanocomposite films with a high-dielectric constant are desired. Herein, we compared the dielectric properties of pure PDMS film, SiC/PDMS and SiC@SiO_2_/PDMS nanocomposite films with the same thickness of 120 μm at the frequency of 1000 Hz and the corresponding results are shown in Fig. S8. Clearly, the addition of SiC whiskers with the mass fraction of 7 wt% slightly decreases the dielectric constant of the pure PDMS film by 3.1% from 2.26 to 2.19, while the dielectric constant of the pure PDMS film increases by 25.2% from 2.26 to 2.83 by doping the SiC@SiO_2_ whiskers of 7 wt%. Additionally, SiC whiskers increase the dielectric loss of the pure PDMS film from 0.0037 to 0.0043, while SiC@SiO_2_ whiskers can reduce its dielectric loss to 0.0023, which increases and decreases 16.2 and 37.8%, respectively. The dielectric constant of the composite film increases as the doping concentrations of SiC@SiO_2_ whiskers increase, while the dielectric loss decreases with the increase in the SiC@SiO_2_ content (Fig. S9). The reason for the decrease in the dielectric constant induced by SiC whiskers is the formation of a semiconductive network. The high-concentration SiC whiskers will contact or overlap each other in the PDMS matrix, leading to an increase in the strong electron conduction to reduce the dielectric constant and increase dielectric loss. However, the SiO_2_ shell layer of SiC@SiO_2_ whiskers has an excellent insulating property, which can effectively inhibit the migration of free carriers, thereby increasing the dielectric constant and reducing the dielectric loss of the SiC@SiO_2_/PDMS nanocomposite films. Based on the high-dielectric constant and the low-dielectric loss SiC@SiO_2_/PDMS nanocomposite film with superhydrophobic Al plate, a contact-separation mode TENG was fabricated. The corresponding working mechanism of the TENG with contact-separation mode during a cyclic four-step process is shown in Fig. 3a. Specifically, the upper and bottom triboelectric layers first contact with each other driven by a linear motor (step-i), thereby generating opposite charges on the surfaces (triboelectrification). In this process, the superhydrophobic layer is easier to lose electrons and shows the positive electric property while the SiC@SiO_2_/PDMS layer is the opposite, which attributes to the difference of electronegativity. When the two triboelectric layers gradually separate from each other, the electrons migrate from the bottom Al electrode to the upper Al electrode and form the electrical current through an external circuit (step-ii) because of electrostatic induction. Two triboelectric layers are completely separated and both charges in upper and bottom electrodes are in balance with that of the corresponding triboelectric layers (step-iii). There is no current in an external circuit once two triboelectric layers are gradually close to each other because the electrostatic balance is broken to induce electrons that are removed from the upper Al electrode to the bottom Al electrode (step-iv). The generated current during step-ii and step-iv is in opposite directions, suggesting the alternative electric performance of the TENG. When two triboelectric layers contact again (step-i), no charge can be generated on two electrodes. The cyclic current signal is also illustrated qualitatively in the inset of Fig. 3a. To better understand and analyze the potential changes of two triboelectric layers induced by charge transfer between two electrodes, we simulate the electrode potential distribution caused by the charge change during the triboelectric layer surface separation by COMSOL (Software for Multiphysics Simulation) software as shown in Fig. 3b. Here, three main potential distributions are simulated in the triboelectrification processes as an ancient natural phenomenon of electricity generation and migration, reflecting different physical properties of materials originated from Maxwell’s displacement current [42].

**Fig. 4:**
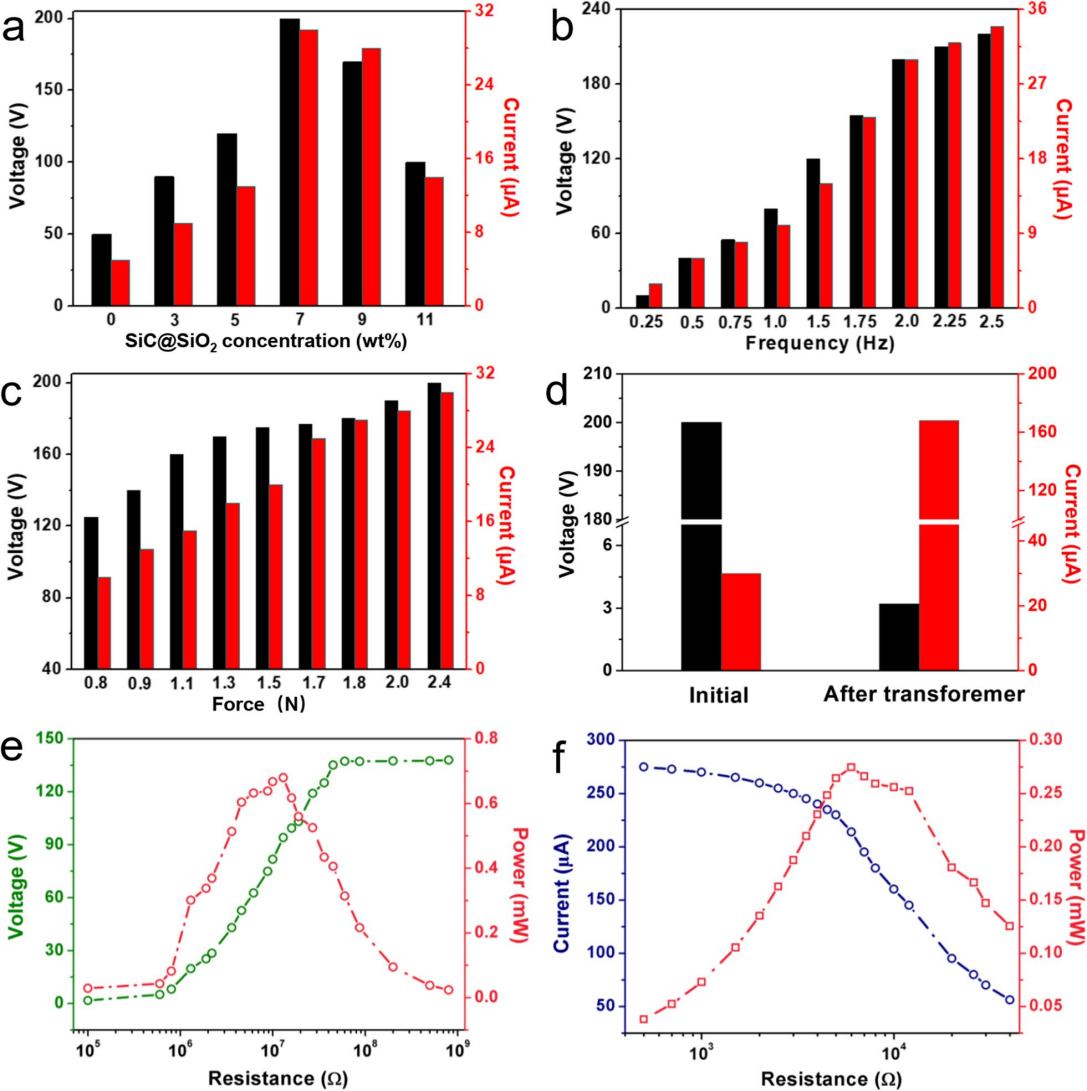
**a** Measured output voltages and currents of the TENG by using SiC@SiO_2_/PDMS composite films with different doping concentrations from 0 to 11 wt%. **b** Measured output voltages and currents of the TENG at different frequencies from 0.25 to 2.5 Hz. **c** Measured output voltages and currents of the TENG under different forces from 0.8 to 2.4 N. **d** Measured output voltages and currents of the TENG before and after using a transformer. **e** Measured output voltages of the TENG under the different loading resistances and the corresponding output powers before using a transformer at a constant force of 2.4 N and a frequency of 2 Hz. **f** Measured output currents of the TENG under the different loading resistances and the corresponding output powers after using a transformer at a constant force of 2.4 N and a frequency of 2 Hz

To evaluate the output performance of the TENG, the influence of various SiC@SiO_2_ doping concentrations, working frequencies and forces were systematically investigated. Initially, the working frequency and force of the linear motor are fixed at 2 Hz and 2 N, respectively and six weight fractions of SiC@SiO_2_ nanowhiskers in a PDMS matrix were selected, including 0, 3, 5, 7, 9, and 11 wt%. Figure S10a and b record the time-dependent voltage and current variation curves and the average peak voltages/currents variation with different concentrations of SiC@SiO_2_ nanowhiskers were plotted as shown in Fig. 4a. Distinctly, both peak voltage and current increase first with the increase in SiC@SiO_2_ concentrations from 0 to 7 wt%, then decrease as the concentration of SiC@SiO_2_ over 7 wt%. The reason may be that the surface roughness of the composite film gradually increases with the increase in doping concentrations of SiC@SiO_2_ nanowhiskers. However, when the doping concentration exceeds 7 wt%, the contact area of two triboelectric materials gradually decreases, resulting in a decline in the output performance of the device. The peak voltage and current of the TENG with the PDMS layer of 7 wt% can reach 200 V and 30 μA, corresponding to the increase by ~ 300 and 500% as compared with the PDMS layer without the SiC@SiO_2_ nanowhiskers, respectively. Therefore, the addition of 7 wt% concentration is the optimal selection for the following investigation. Once the working frequencies of the linear motor from 0.25 to 2.5 Hz, the output voltages and current gradually increase from 10 V/3 μA to 220 V/34 μA as shown in Fig. S10c and d, with which Fig. 4b shows the corresponding statistical results. In addition, when the working frequency is over 2 Hz, there is a relatively small increase in electrical signals. To facilitate comparison and adjustment in subsequent tests, the working frequency of the linear motor is controlled at 2 Hz as shown in Fig. S10e and f. The corresponding statistical results are also shown in Fig. 4c. The increase in pressure by a linear motor can enhance the electrical output of the TENG. Once the force increases from 0.8 to 2.4 N, the generated voltage and current increase by 60 and 200% from 125 V/10 μA to 200 V/30 μA, respectively. Note that the maximum transfer charge of the TENG is 54 nC, as presented in Fig. S11, and the calculated charge density is approximately 34 μC/m^2^. Generally speaking, TENG shows the electrical output characteristics of high voltage and low current. To obtain a higher current and a certain voltage to meet the working requirements of some small-power electronic devices in practical application, we further compared the output performance of the initial electric signals and the improved electric signals using a transformer with coil construction and a rectifier as shown in Figs. 4d and S12, for which the TENG with the frequency of 2 Hz and the force of 2.4 N were operated. The peak voltage and current were modified from 200 V/30 μA to 3.2 V/168 μA using the transformer and the rectifier, which are exactly what we expected with the output voltage and current of ~ 3 V and ~ 160 μA. Furthermore, the relationship between impedance and maximum output power of the TENG before and after transformation and rectification were investigated at the working frequency and the pressure of the linear motor at 2 Hz and 2.4 N as shown in Fig. 4e and f. With the increase in the external resistance, the voltage shows a rising trend while the output power shows a trend of first increasing and then decreasing behaviors. When a 1.3 MΩ resistor is externally loaded, the TENG produces the maximum power of 0.68 mW with the corresponding output voltage of 94 V at this time (Fig. 4e). Note that the measured output current of the TENG decreases with the increase in the load impedance using a transformer and the maximum output power is approximately 0.27 mW under an external loading resistance of 6 kΩ as shown in Fig. 4f. The power of TENG is reduced due to the electrical loss of the transformer. Additionally, the voltage and the output power of the TENG at the frequency of 1  and 2.5 Hz were also tested as shown in Fig. S13a and S13b. At a low frequency, the TENG delivers the maximum power of 0.19 mW corresponding to the external resistance of 1.3 MΩ, while the maximum power is 2.24 mW with the external resistance of 0.95 MΩ at a high frequency, further confirming the opinion that the increase in the working frequency will increase the electrical output performance of the TENG.

**Fig. 5:**
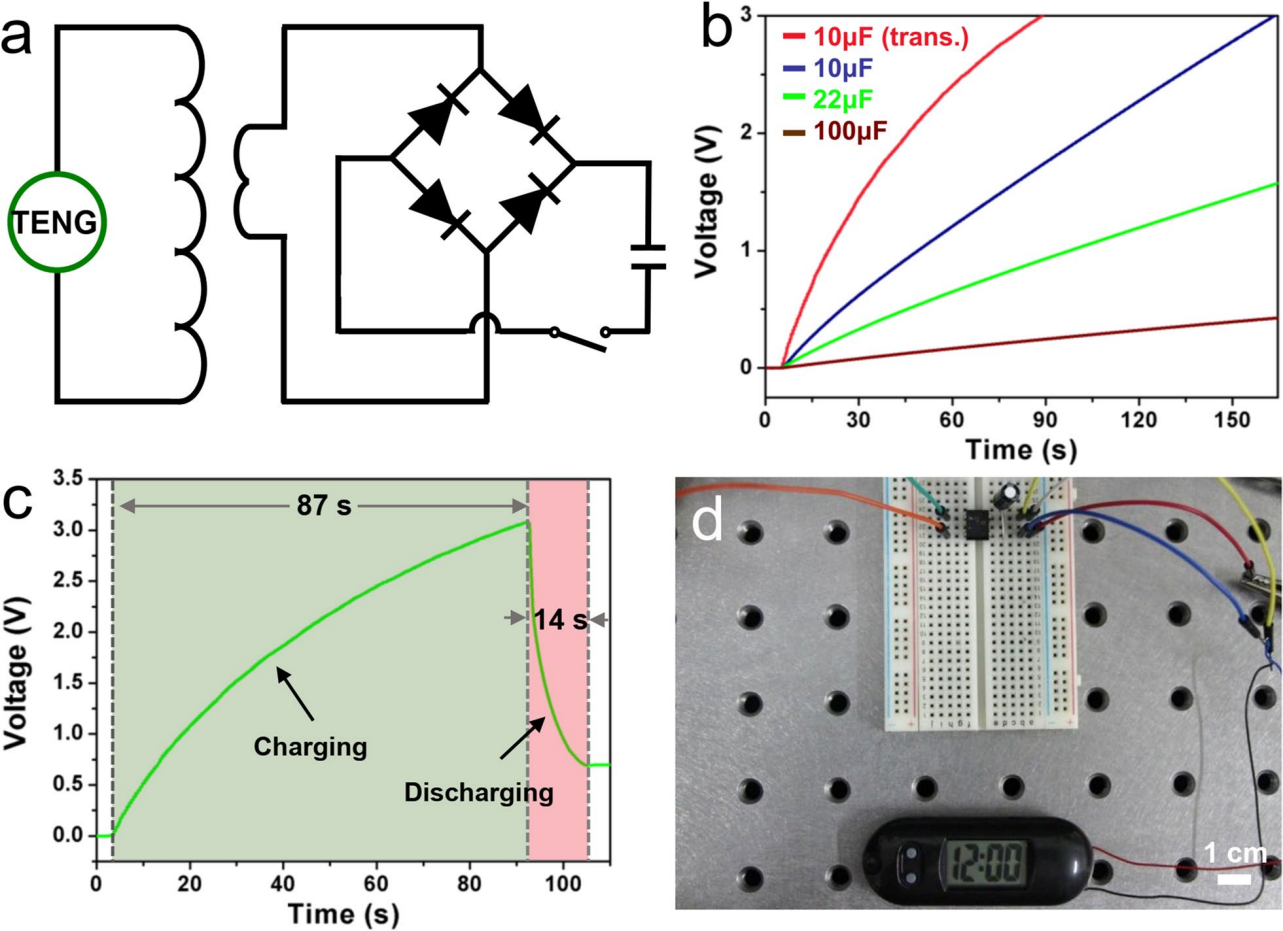
**a** Equivalent circuit of the TENG connected to the external circuit through a transformer and a rectifier. **b** Charging curves of the different capacitors via the TENG. **c** Charging and discharging voltage–time curve of a 10 μF capacitor used as storage and power supply unit by using the TENG with a transformer and a rectifier to power an electronic watch. **d** Photograph of an electronic watch being powered by the TENG

To demonstrate the practical application of the fabricated TENG in daily life, an effective method is to store the electricity generated by the TENG to a commercial capacitor. Figure 5a shows the management circuit diagram for a single TENG, which can be charged to a capacitor by the transformer and the rectifier. Based on this circuit, the charging curves of three different capacitors are shown in Fig. 5b. 22 and 100 μF capacitors can be charged from 1.57 and 0.43 V within 165 s connected by a rectifier, respectively, revealing that the smaller the capacitance is, the faster the charging speed is. To compare the charging speed of two charging methods to TENG, the 10 μF capacitor is respectively charged using a rectifier and the combination of a transformer and a rectifier. It can be clearly observed from blue line and red line as shown in Fig. 5b that two circuits charge take 165 s and 90 s, respectively, to charge the 10 μF capacitor to 3 V. Furthermore, Fig. 5c exhibits charging and discharging curves of the 10 μF capacitor. After charging to 3 V in 87 s as a power source, the 10 μF capacitor can drive an electronic watch to work with ~ 14 s, which reflects the potential application value of the TENG in daily lives as shown in Fig. 5d.

## Conclusions

A high-performance TENG consisting of the superhydrophobic Al plate and the high-dielectric SiC@SiO_2_/PDMS nanocomposite films as positive and negative triboelectric layers was demonstrated, respectively. The output performance of the TENG can be greatly increased after mixing SiC@SiO_2_ core–shell nanoparticles into the PDMS film, which ascribes to the increase in dielectric permittivity and the decrease in dielectric loss of the PDMS composite film because of the high electric insulation among SiC@SiO_2_ nanoparticles. The output voltage/current of the TENG can be significantly increased from 50 V/5 μA to 200 V/30 μA after the addition of 7 wt% SiC@SiO_2_ core–shell nanoparticles into the PDMS film compared with the pure PDMS triboelectric layer. Moreover, the excellent hydrophobicity of the positive and negative triboelectric layers leads to the high self-cleaning performance of the TENG. Meanwhile, we have demonstrated the practical application of the TENG to charge a commercial capacitor and to power an electronic watch continuously. This work can provide an effective way for the rational design of high-performance TENGs by regulating the dielectric properties of triboelectric materials based on core–shell nanowhiskers.

## Supplementary Information

Supplementary file

## Data Availability

All data supporting the conclusions of the article are included within the article.
