# Supplementary material for: Rational design on high-performance triboelectric nanogenerator consisting of silicon carbide@silicon dioxide nanowhiskers/polydimethylsiloxane (SiC@SiO2/PDMS) nanocomposite films
Source: Discov Nano. 2023 Apr 21;18(1):69. doi: 10.1186/s11671-023-03822-8 (PMC10409695; doi:10.1186/s11671-023-03822-8)
Supplement: Supplementary file 1 — Supplementary file [file 11671_2023_3822_MOESM1_ESM.docx]

**Supporting Information**

**Rational Design on High-Performance Triboelectric Nanogenerator Consisting of Silicon Carbide@Silicon Dioxide Nanowhiskers/Polydimethylsiloxane (SiC@SiO_2_/PDMS) Nanocomposite Films**

Kun Zhao^1,^*, Wanru Sun^1^, Suixin Li^1^, Zhenhua Song^1^, Ming Zhong^1^, Ding Zhang^2^, Bing-Ni Gu^3,5,6,7^, Ming-Jin Liu^3,5,6,7^, Hao Fu^4^, Hongjie Liu^4^, Cheng Meng^8^ and Yu-Lun Chueh^3,5,6,7,^*

^1^State Key Laboratory of Advanced Processing and Recycling of Nonferrous Metals, Lanzhou University of Technology, Lanzhou 730050, P. R. China

^2^School of Materials Science and Engineering, National Institute for Advanced Materials, Nankai University, Tianjin 300350, P. R. China

^3^Department of Materials Science and Engineering, National Tsing Hua University, Hsinchu 30013, Taiwan

^4^School of Chemistry and Chemical Engineering, Guangxi University, Nanning 530004, P. R. China

^5^Colleage of Semiconductor Research, National Tsing-Hua University, Hsinchu, 30013, Taiwan

^6^Department of Physics, National Sun Yat-Sen University, Kaohsiung 80424, Taiwan

^7^Frontier Research Center on Fundamental and Applied Sciences of Matters, National Tsing Hua University, Hsinchu 30013, Taiwan

^8^Jiangxi Province Key Laboratory of Polymer Micro/Nano Manufacturing and Devices, School of Chemistry Biology and Materials Science, East China University of Technology, Nanchang 330013, P.R. China

*Corresponding authors. E-mail: ylchueh@mx.nthu.edu.tw and zhaokun@lut.edu.cn


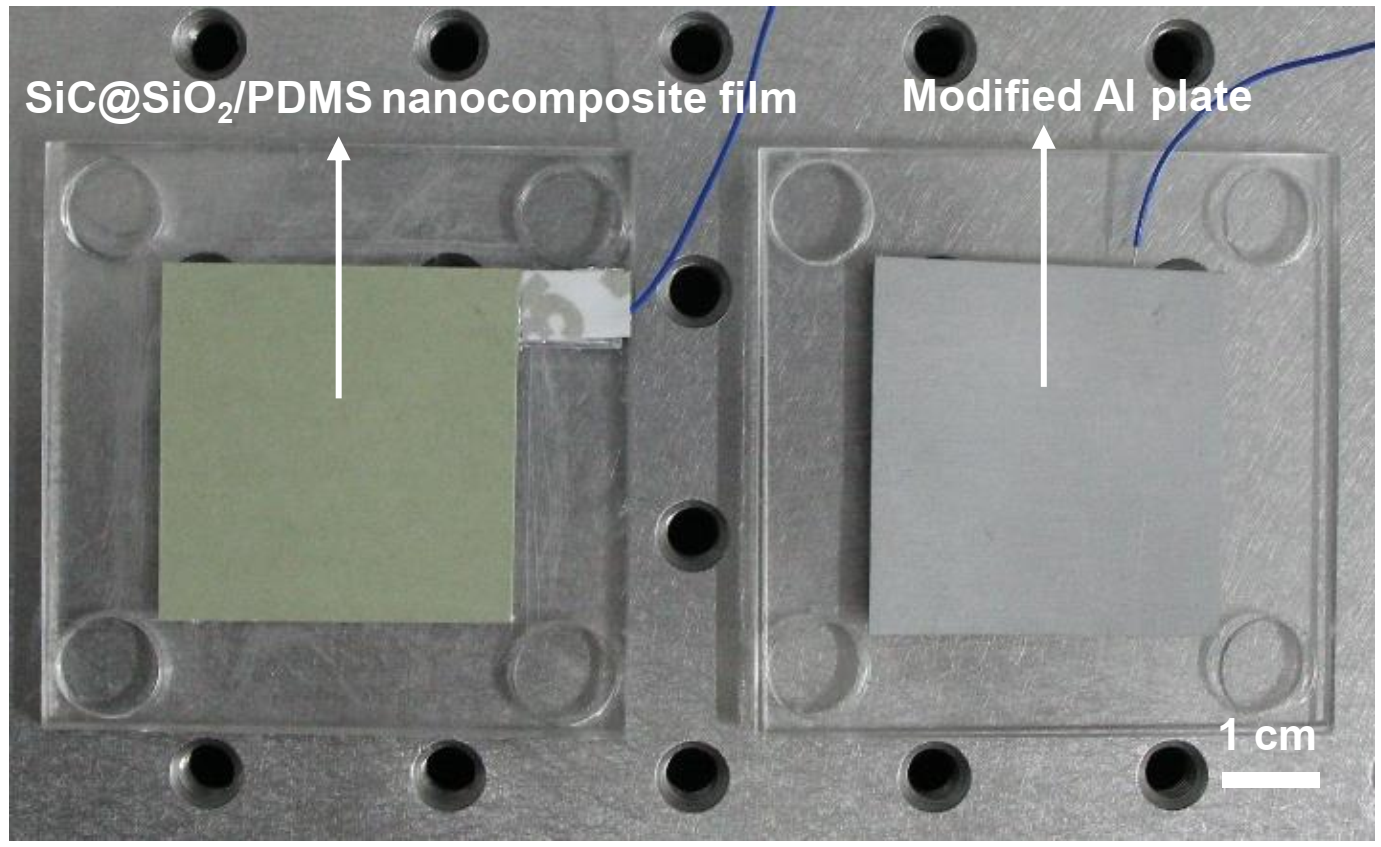


**Fig. S1** A photograph of the main assembly parts for TENG.


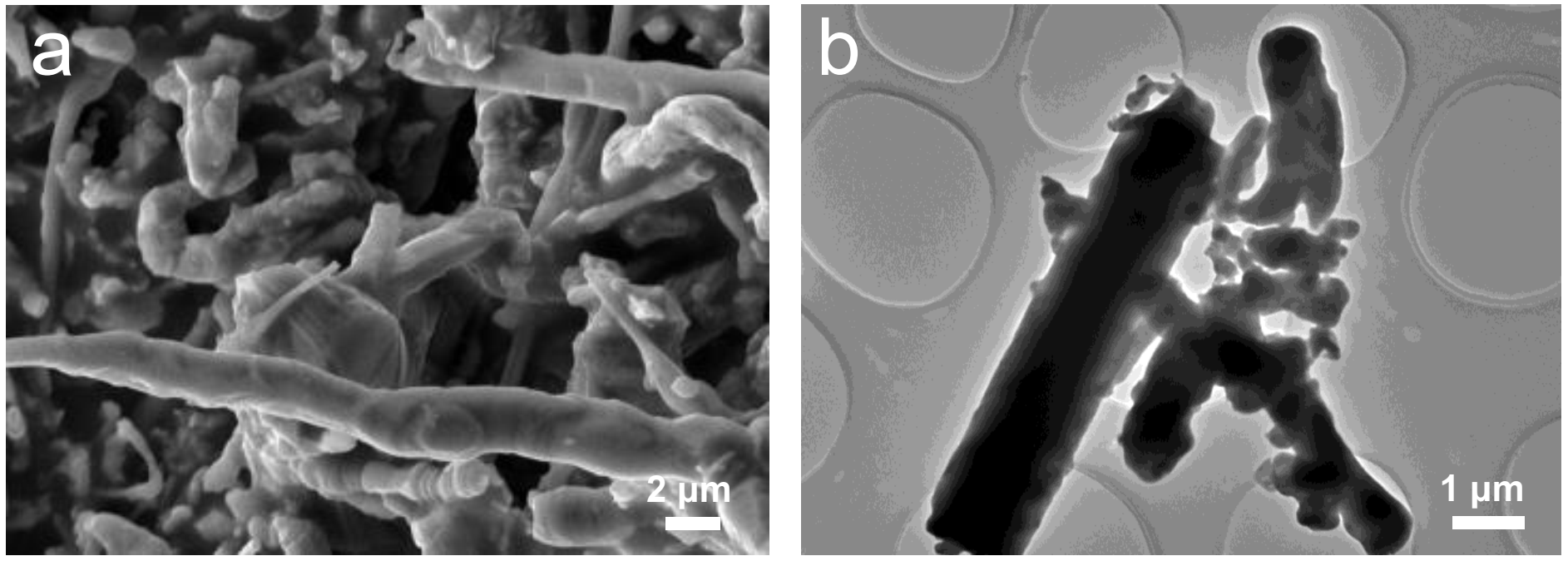


**Fig. S2** (a,b) Low-magnification SEM (a) and TEM (b) images of SiC@SiO_2_ core-shell nanowhiskers.


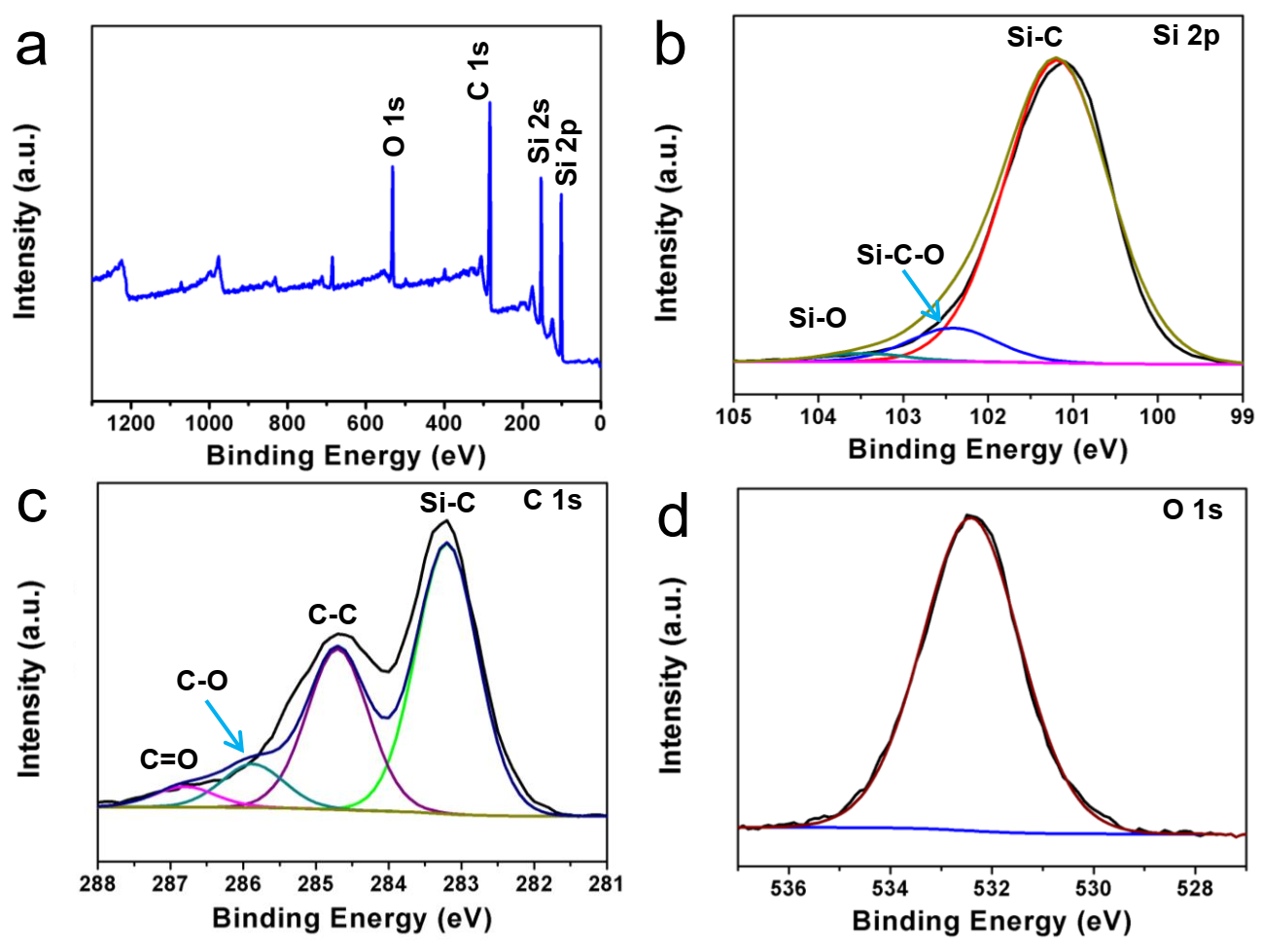


**Fig. S3** (a-c) XPS spectra (a) and high-resolution Si 2p (b), C 1s (c) and O 1s (d) XPS spectra of the SiC

whiskers.


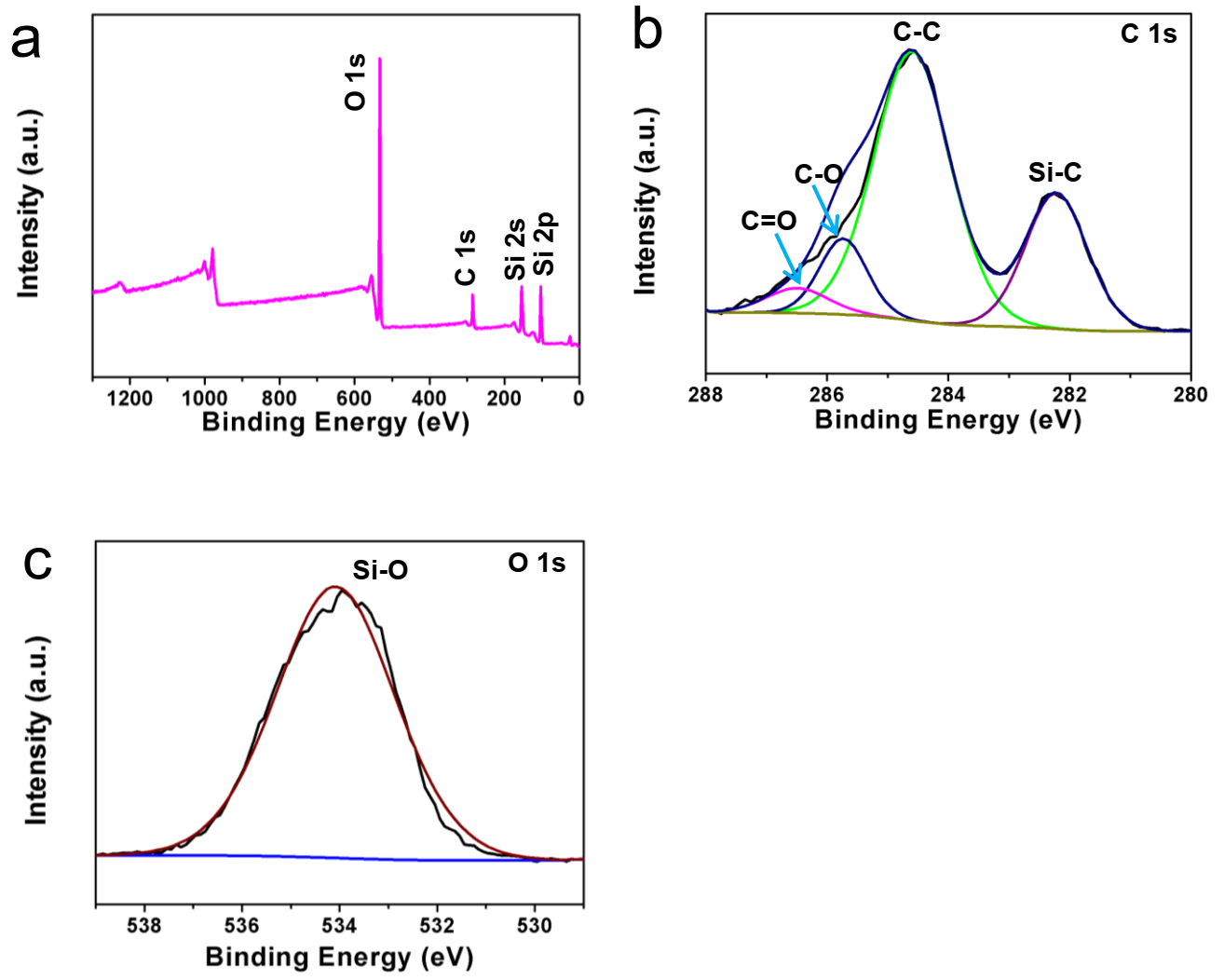


**Fig. S4** (a-c) XPS spectra (a) and high-resolution C 1s (b) and O 1s (c) XPS spectra of the SiC@SiO_2_.


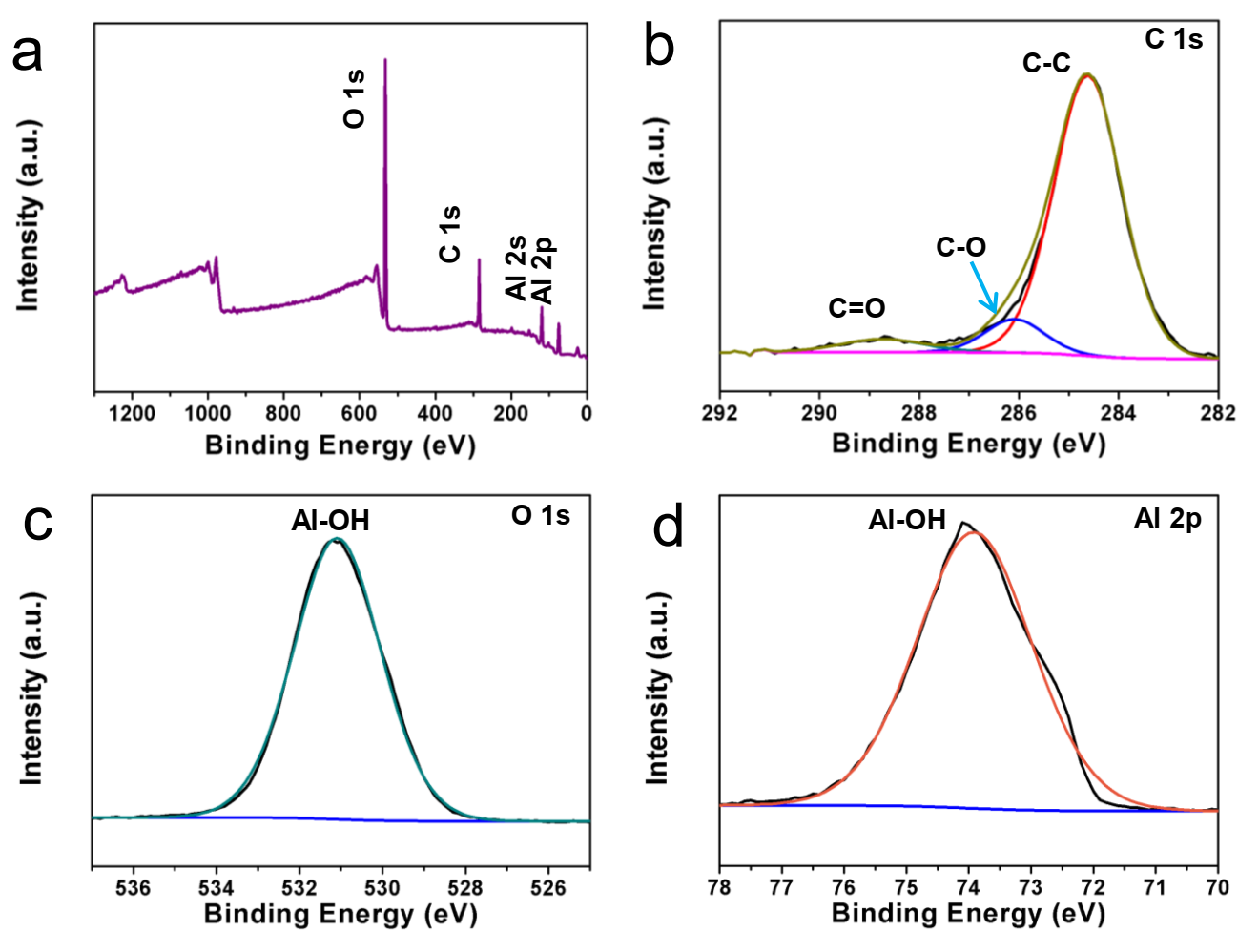


**Fig. S5** (a-d) XPS spectra (a) and high-resolution C 1s (b), O 1s (c) and Al 2p (d) XPS spectra of the etched Al plate.


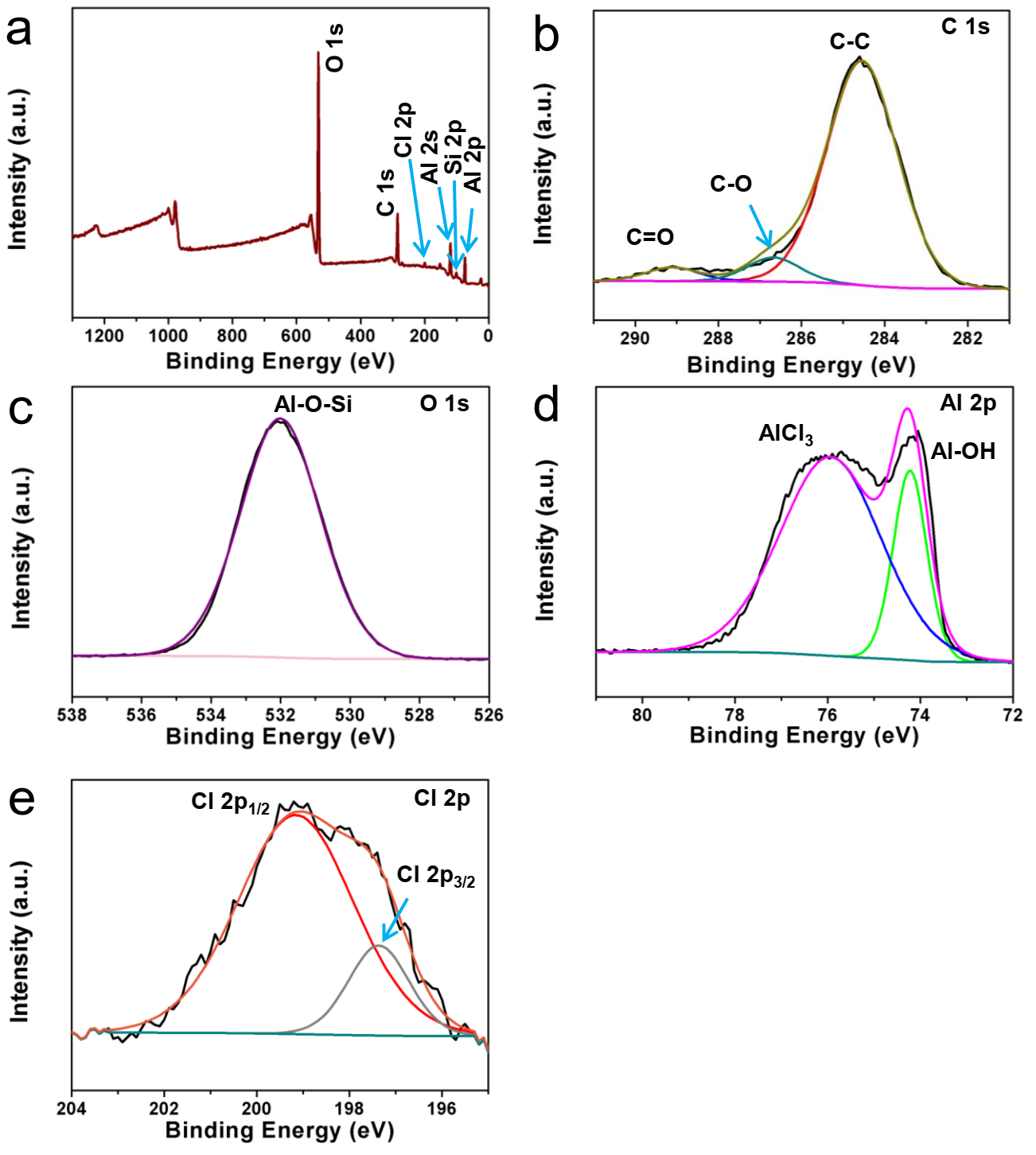


**Fig. S6** (**a-e**) XPS spectra (a) and high-resolution C 1s (b), O 1s (c), Al 2p (d) and Cl 2p (e) XPS spectra of the etched Al plate modified with octadecyltrichlorosilane.


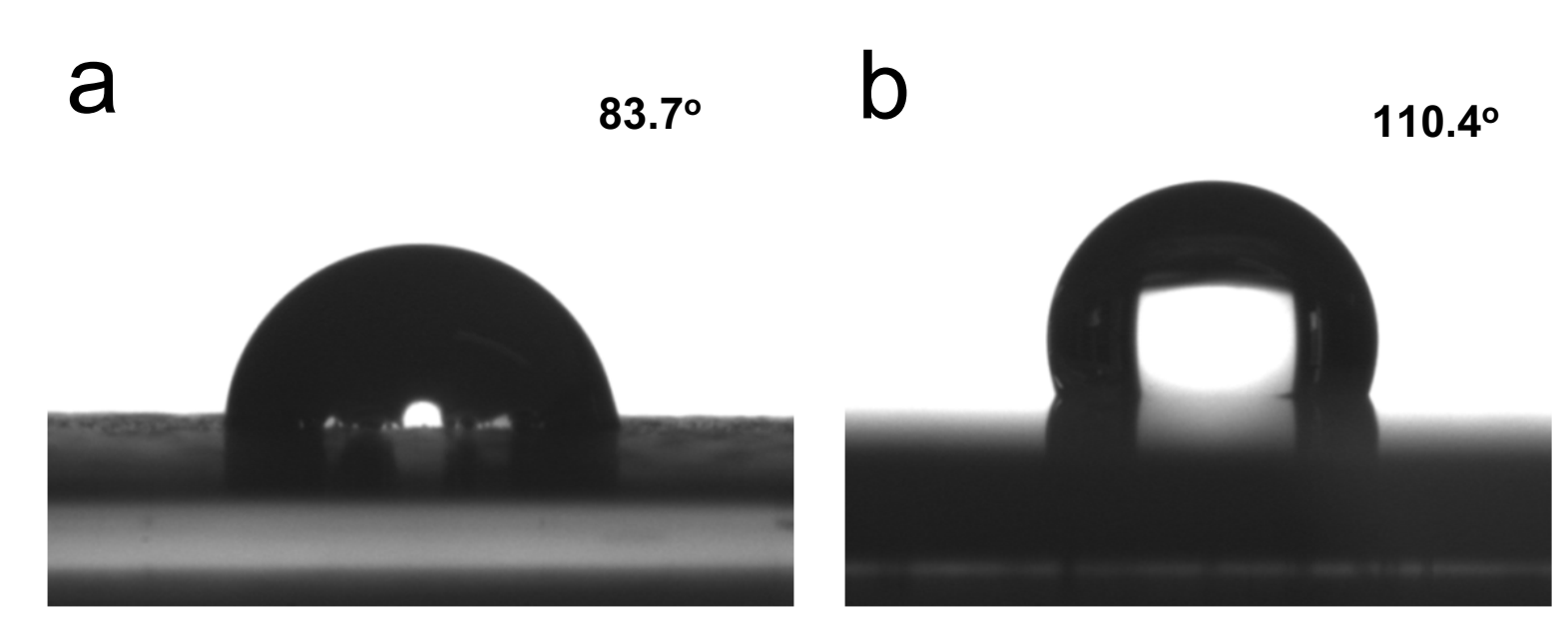


**Fig. S7** (**a,b**) Photographs of a drop of water on the etched Al plate (a) and the pure PDMS film (b).

**
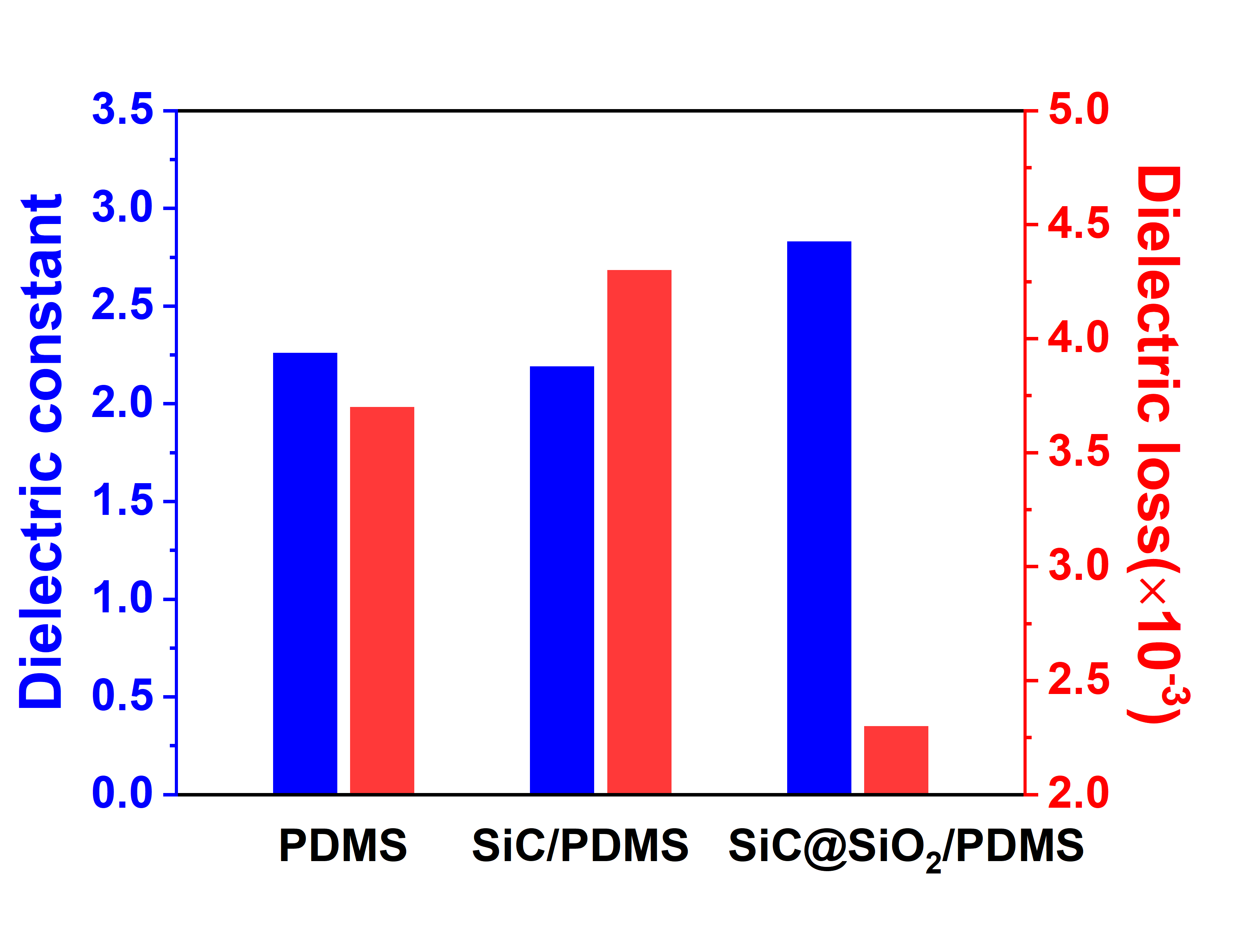
**

**Fig. S8** Dielectric constant and dielectric loss of pure PDMS film, SiC/PDMS and PDMS/SiC@SiO_2_

nanocomposite films with the doping concentration of 7 wt% at the frequency of 1000 Hz.


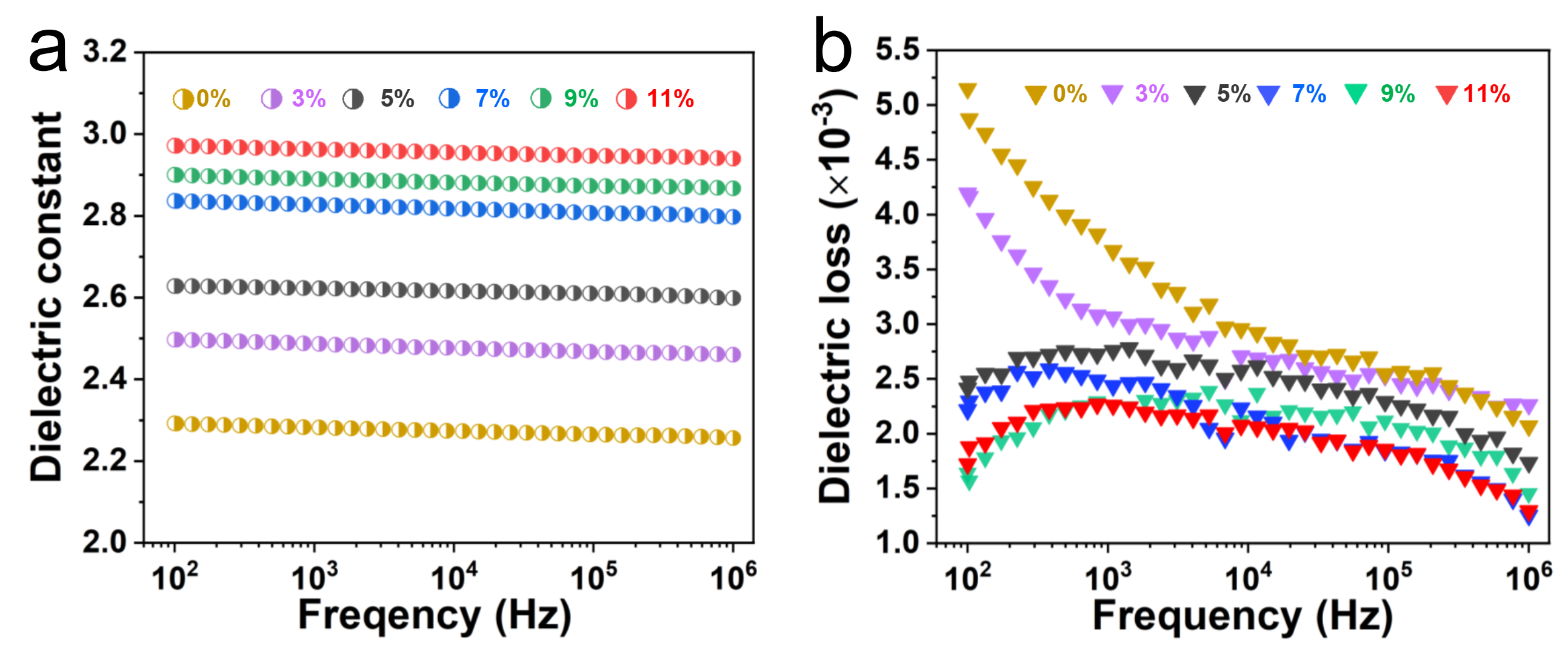


**Fig. S9** (a,b) Frequency dependence of the dielectric constant (a) and dielectric loss (b) of SiC@SiO_2_/PDMS composite films with different doping concentrations of SiC@SiO_2_ nanoparticles.


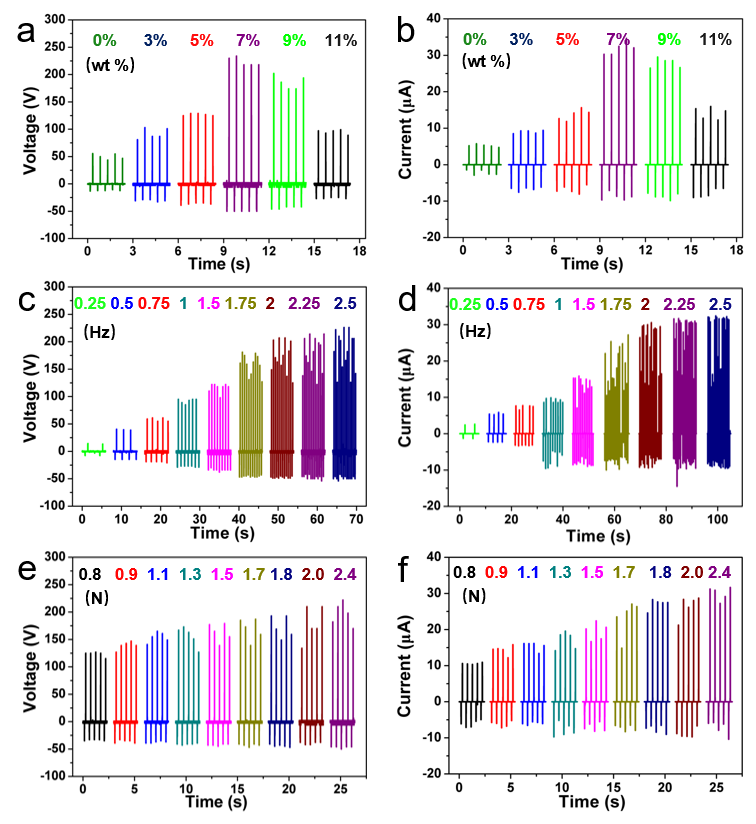


**Fig. S10** (a,b) Measured output voltages (a) and current (b) signals of the TENG by using PDMS/SiC@SiO_2_

nanocomposite films with different doping concentrations. (c,d) Measured output voltage (c) and

current (d) signals of the TENG at different frequencies. (e,f) Measured output voltage (e) and current

(f) signals of the TENG under different forces.

*
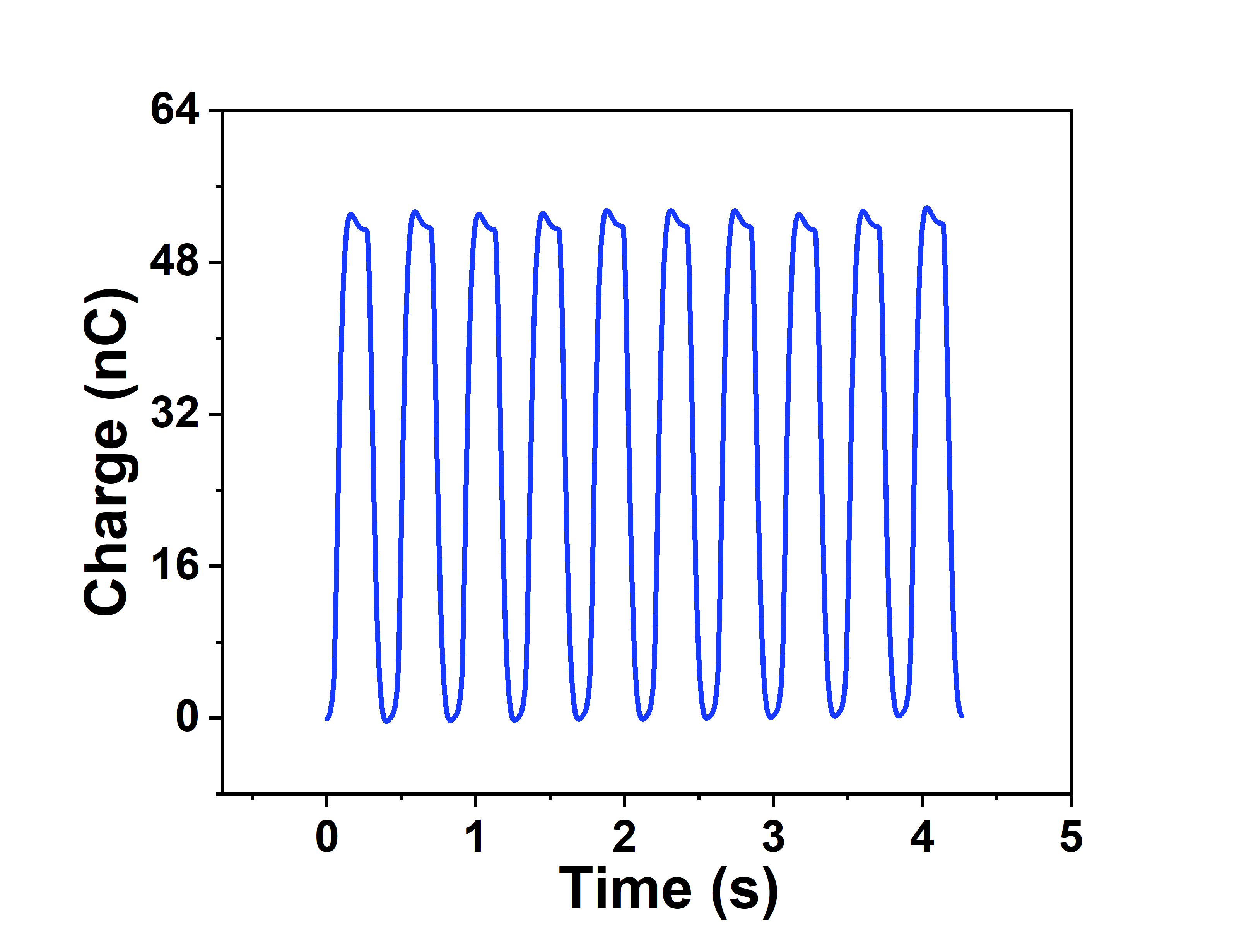
*

**Fig. S11** Measured transfer charge of the TENG.


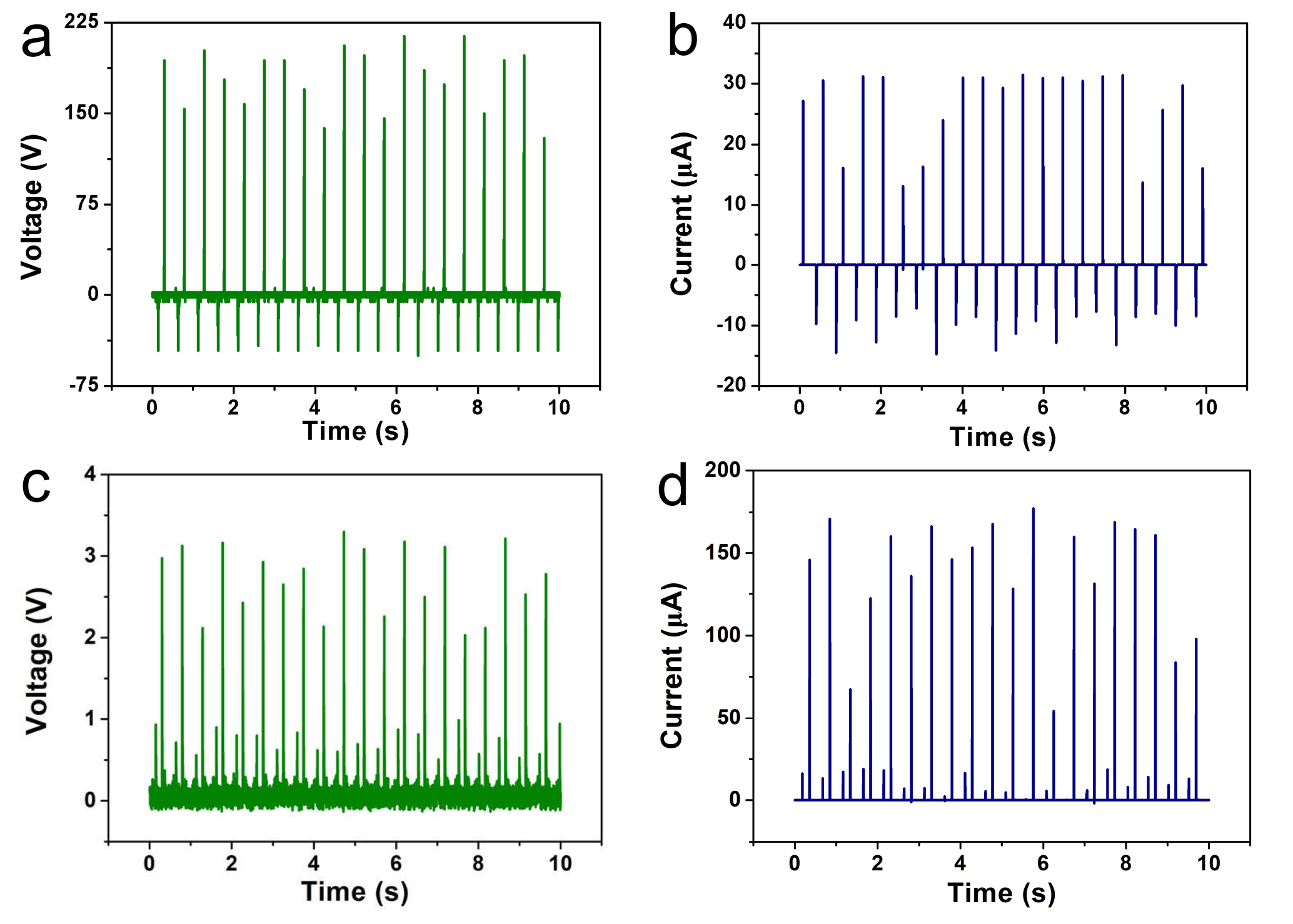


**Fig. S12** (a,b) Measured output voltages (a) and current (b) signals of the TENG at a constant force of 2.4 N and a frequency of 2 Hz. (c,d) Measured voltage (c) and current (d) signals of the TENG by using a transformer and a rectifier at a constant force of 2.4 N and a frequency of 2 Hz.


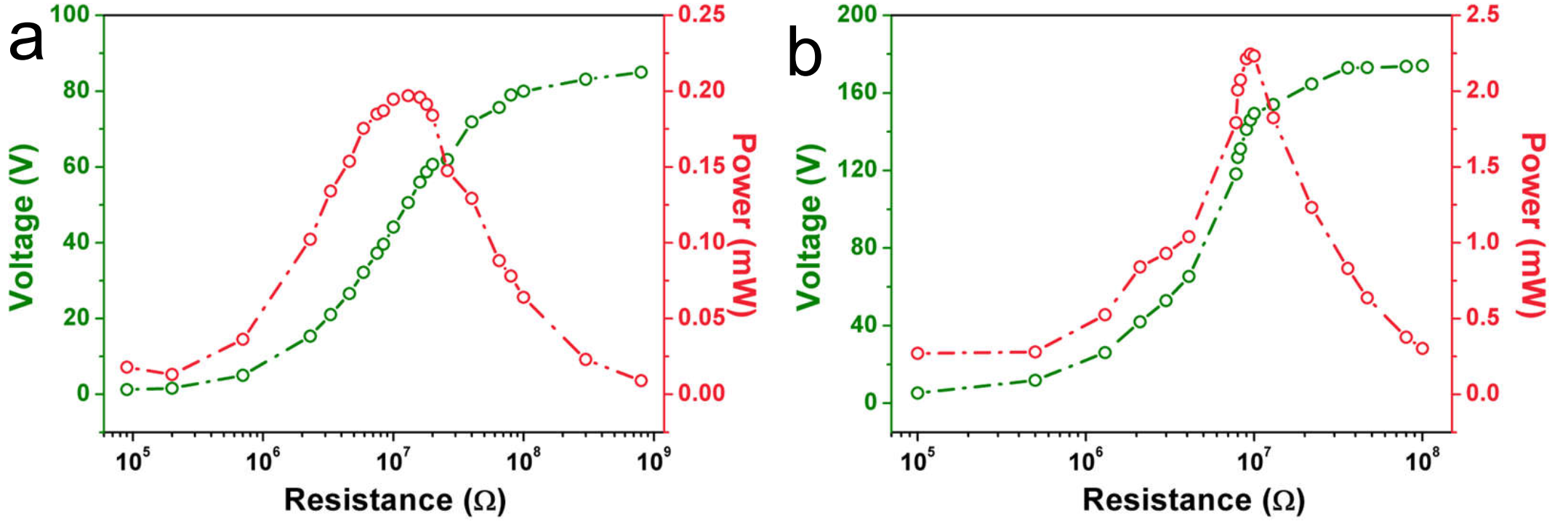


**Fig. S13** (a,b) Measured output voltages of the TENG under the different loading resistances and the

corresponding powers at a constant force of 2.4 N and frequencies of 1 Hz (a) and 2.5 Hz (b).
